# Supplementary material for: Acetyl-CoA-Carboxylase 1-mediated de novo fatty acid synthesis sustains Lgr5+ intestinal stem cell function
Source: Nat Commun. 2022 Jul 9;13:3998. doi: 10.1038/s41467-022-31725-2 (PMC9271096; doi:10.1038/s41467-022-31725-2)
Supplement: Supplementary file 1 — Supplementary Information [file 41467_2022_31725_MOESM1_ESM.pdf]

Supplementary Figure 1

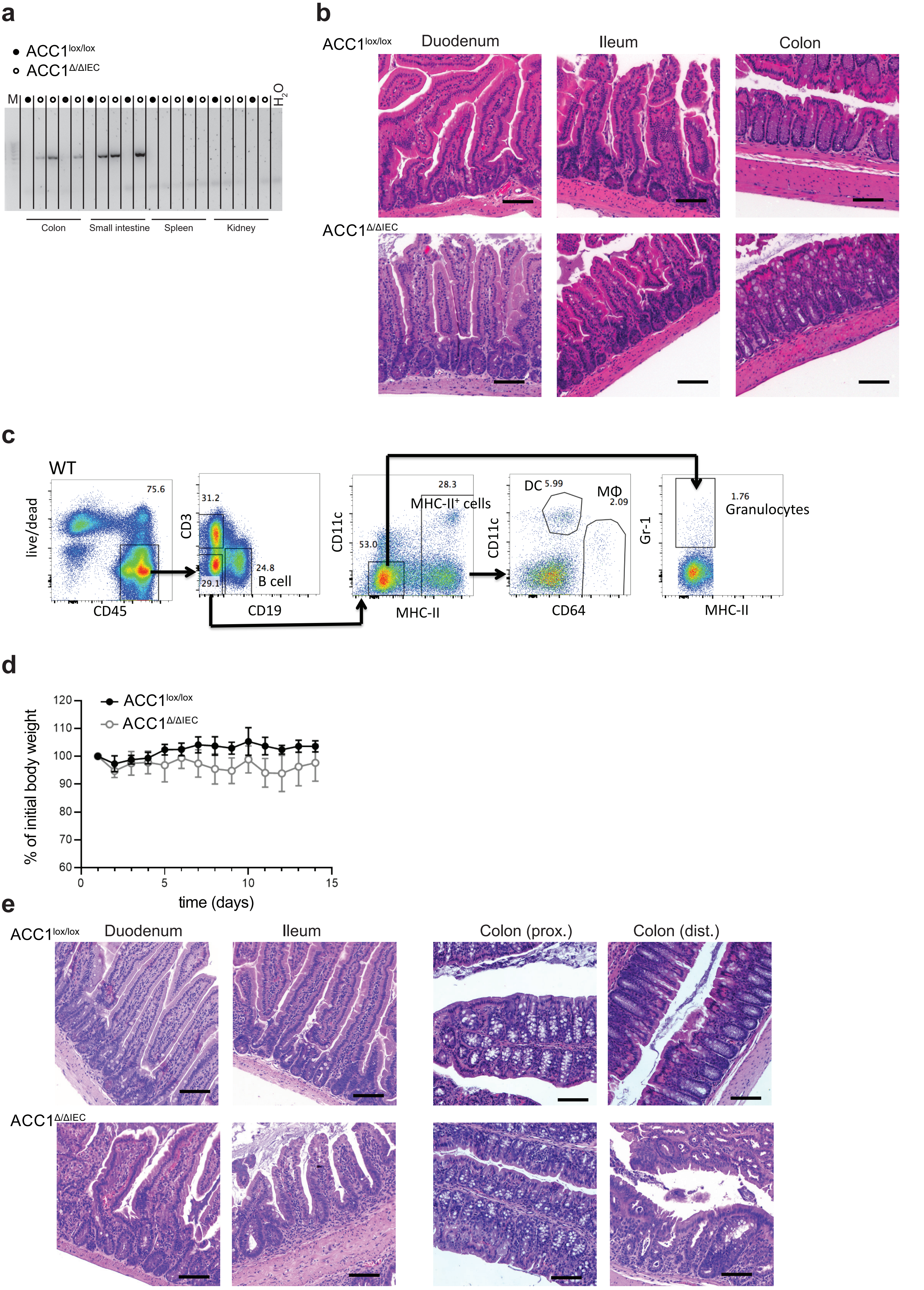

Supplementary Figure 2

a

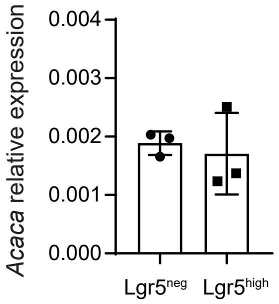

b

Colon

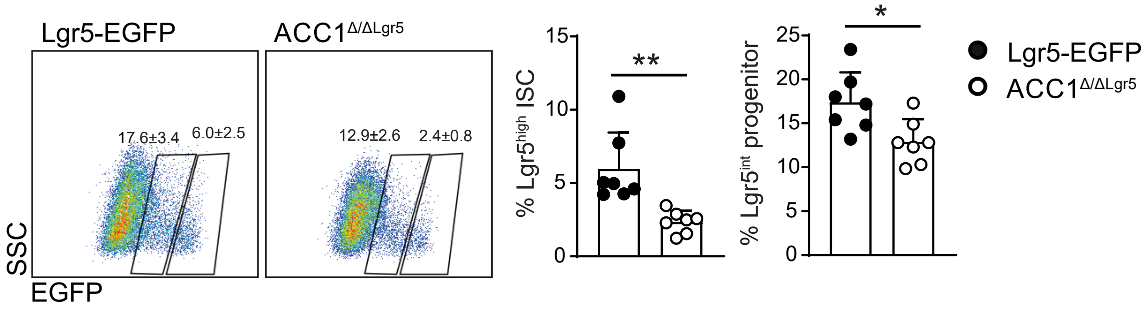

Supplementary Figure 3

a

$ACC1^{lox/lox}$

$ACC1^{\Delta/\Delta IEC}$

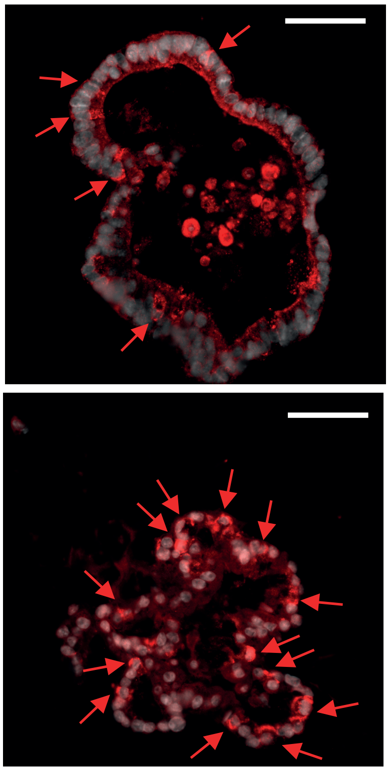

b

ID	Description	enrichmentScore	NES	p.adjust
GO:0098687	chromosomal region	-0,607974339	-2,863431	1,73355E-30
GO:0006281	DNA repair	-0,49278458	-2,270492	4,84893E-27
GO:0000775	chromosome, centromeric region	-0,661747648	-3,039689	1,06732E-25
GO:0007059	chromosome segregation	-0,555916465	-2,683897	2,43235E-25
GO:0009617	response to bacterium	0,595330652	1,8975875	2,08785E-20
GO:0006260	DNA replication	-0,562506056	-2,51423	2,92695E-20
GO:0000819	sister chromatid segregation	-0,627199408	-2,897775	3,94535E-20
GO:0098813	nuclear chromosome segregation	-0,555707194	-2,483841	1,24423E-19
GO:0000776	kinetochore	-0,670907669	-2,975187	1,202E-18
GO:0000070	mitotic sister chromatid segregation	-0,642074836	-2,91719	3,65321E-18
GO:0006261	DNA-dependent DNA replication	-0,661380877	-2,93735	8,60451E-18
GO:0045087	innate immune response	0,55853003	1,7808798	1,55332E-14
GO:0000793	condensed chromosome	-0,581759906	-2,590241	1,87752E-13
GO:0006310	DNA recombination	-0,471943667	-2,153849	2,69928E-13
GO:0071219	cellular response to molecule of bacterial origin	0,666228893	2,0617682	6,26014E-13
GO:0000280	nuclear division	-0,409765891	-1,898609	7,24931E-13
GO:0051673	membrane disruption in other organism	0,919650762	2,3298051	1,09379E-12
GO:0033044	regulation of chromosome organization	-0,415800173	-1,998018	1,17373E-12
GO:0002237	response to molecule of bacterial origin	0,614538985	1,9321626	1,59938E-12
GO:0051983	regulation of chromosome segregation	-0,654254164	-2,827963	1,64409E-12
GO:0022613	ribonucleoprotein complex biogenesis	-0,39944072	-1,75653	1,64409E-12
GO:0071216	cellular response to biotic stimulus	0,641381768	1,9951683	2,08695E-12
GO:0032496	response to lipopolysaccharide	0,623197681	1,9554965	2,12364E-12
GO:0071222	cellular response to lipopolysaccharide	0,670586536	2,0715902	2,12364E-12
GO:0140097	catalytic activity, acting on DNA	-0,504748005	-2,352855	6,6697E-12
GO:0048285	organelle fission	-0,395645744	-1,818703	7,72254E-12
GO:0006302	double-strand break repair	-0,486889269	-2,29122	1,53542E-11
GO:0044391	ribosomal subunit	-0,50999508	-2,355873	1,53542E-11
GO:0019730	antimicrobial humoral response	0,775763973	2,2189861	1,53542E-11
GO:0006954	inflammatory response	0,533844811	1,7034629	4,33348E-11

c

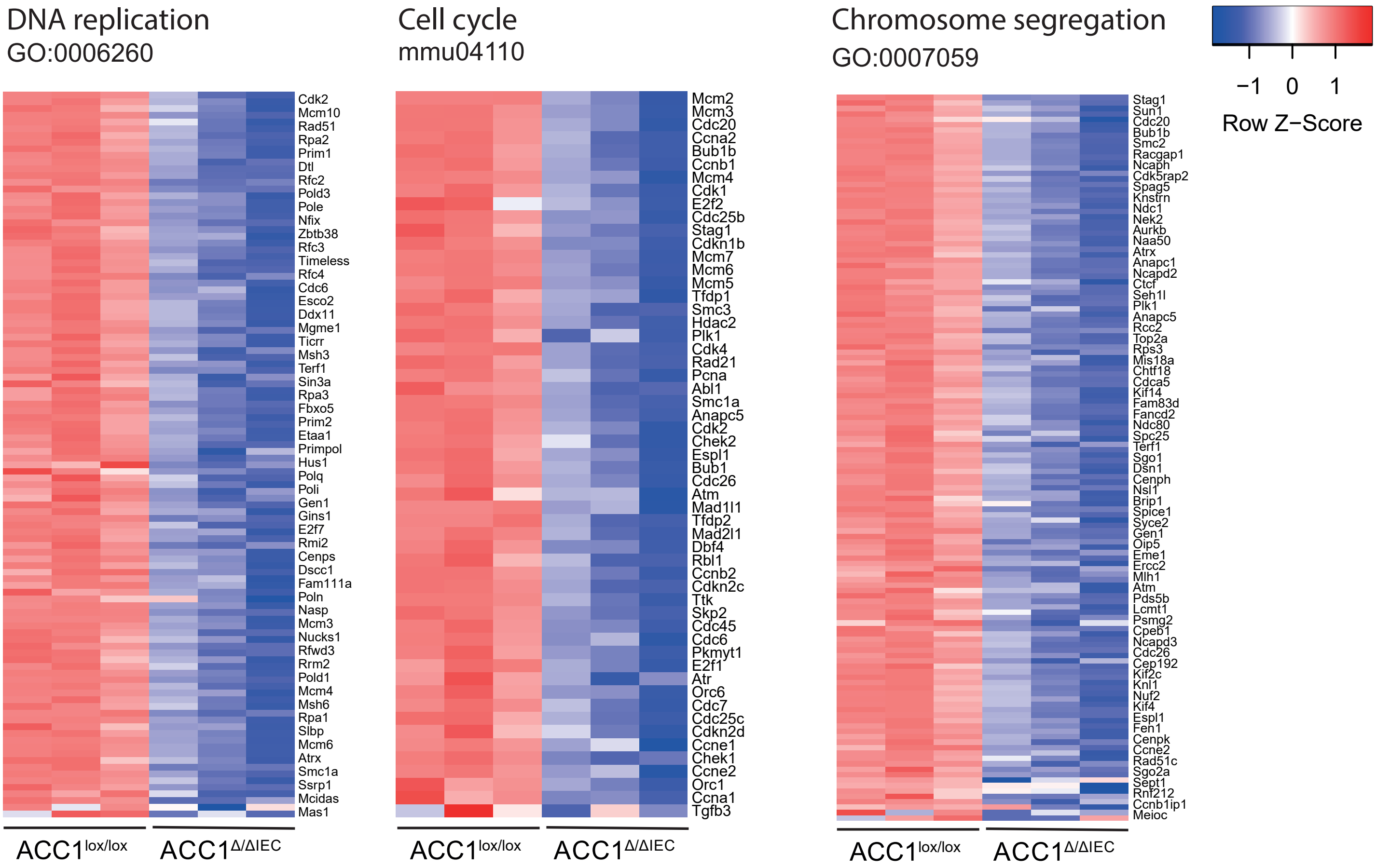

d

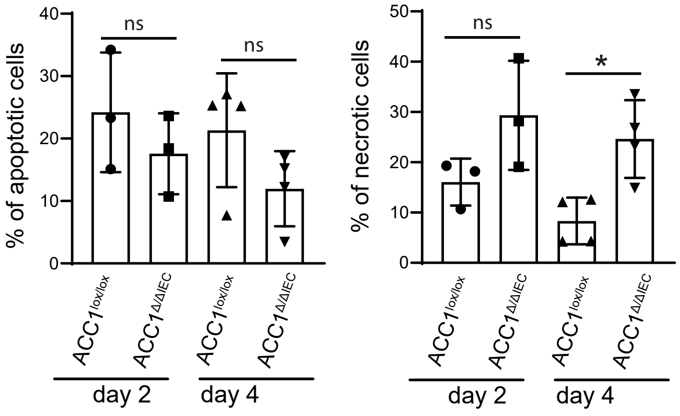

Supplementary Figure 4

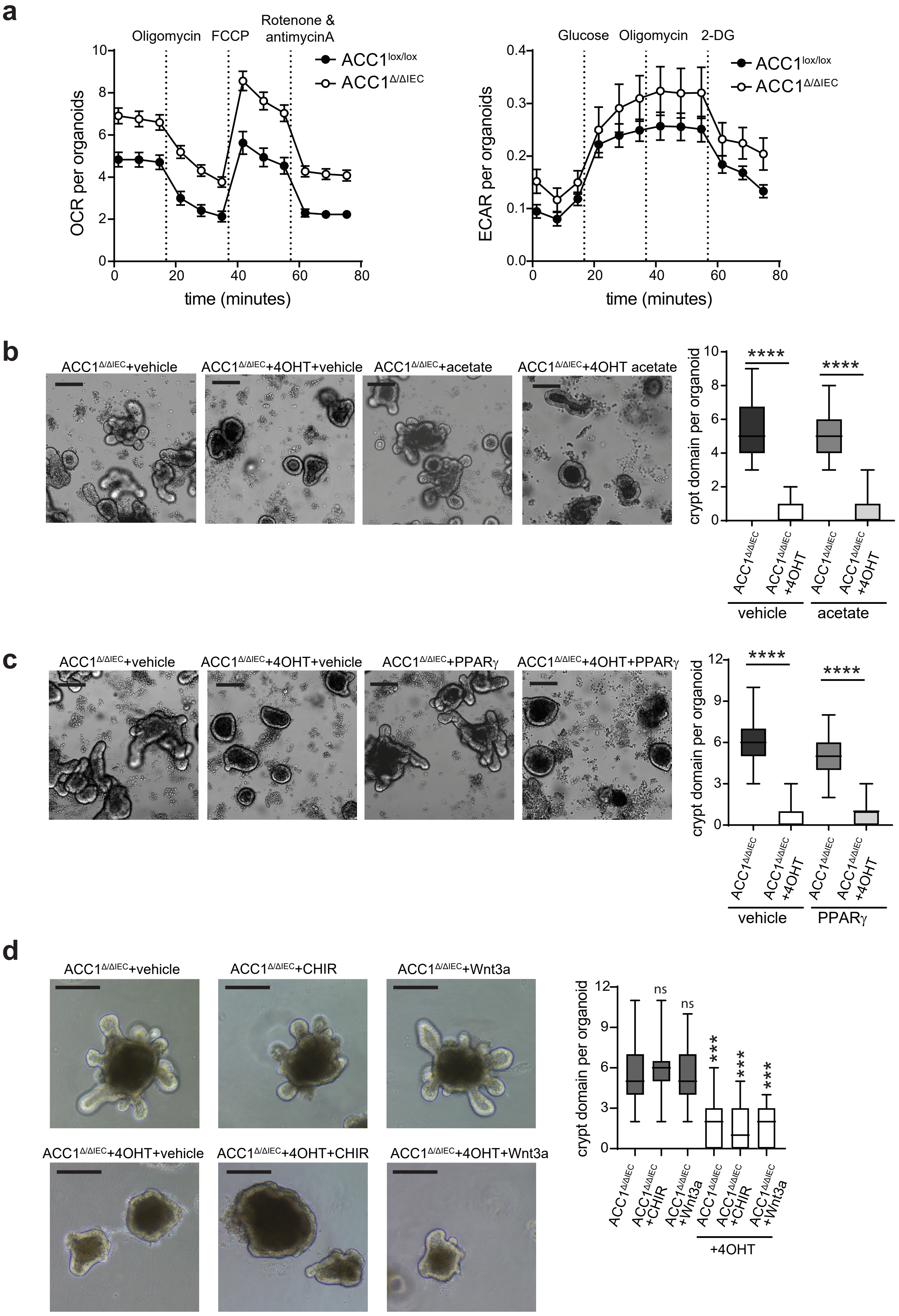

Supplementary Figure 5

a

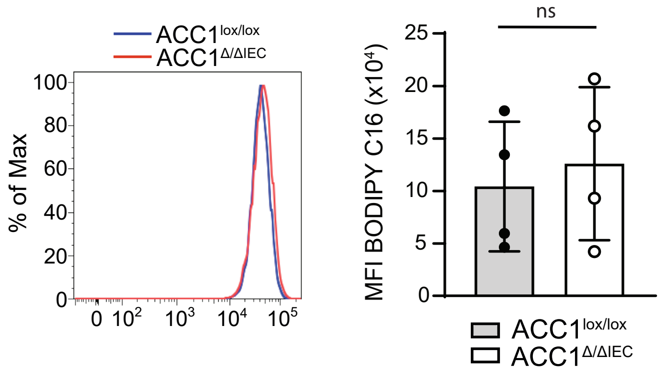

b

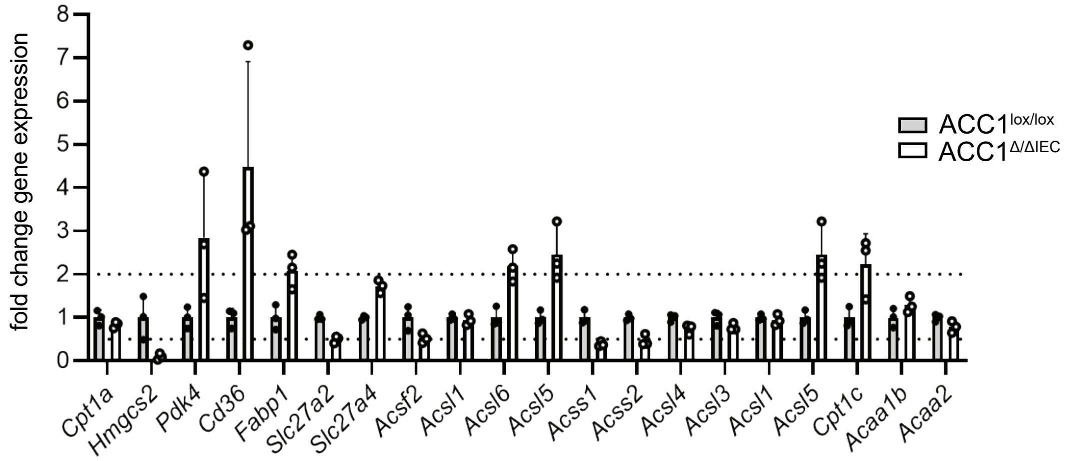

Supplementary Figure 6

a

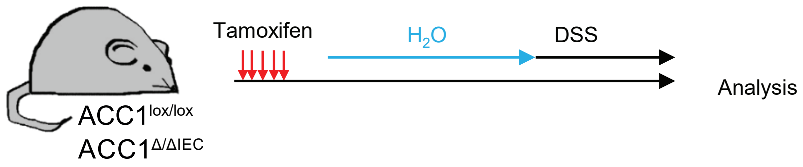

b

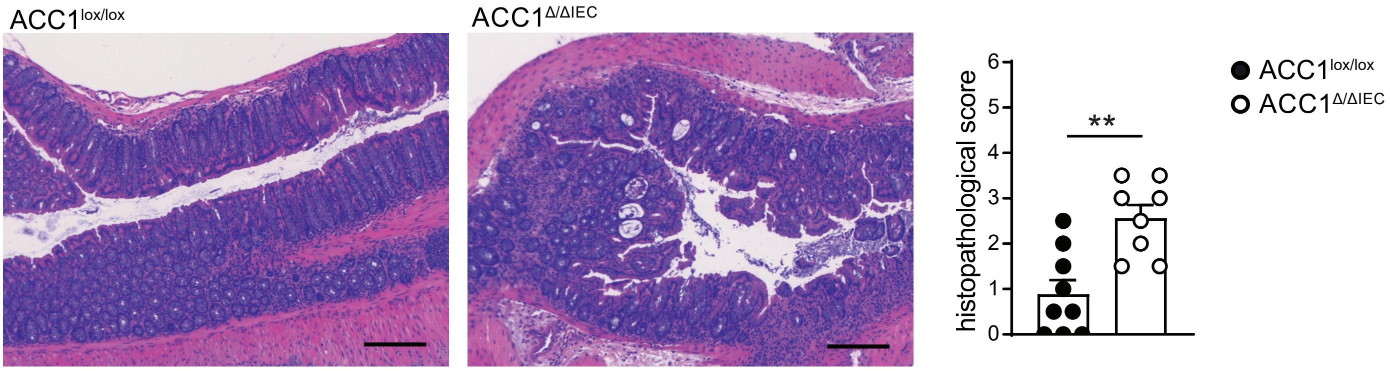

SUPPLEMENTARY FIGURE LEGENDS

Supplementary Figure 1: Specific ACC1 deletion in the intestinal epithelium of ACC1$^{\Delta/\Delta IEC}$ mice has long term effects on the intestinal crypt architecture. (**a**) ACC1$^{lox/lox}$ and ACC1$^{\Delta/\Delta IEC}$ mice were treated with tamoxifen for 5 consecutive days. DNA was extracted from colonic and small intestinal IEC, as well as from the spleen and the kidney one day after the last tamoxifen injection. PCR-based genotyping was performed with primers specifically recognizing the deletion of exon 22 of the *Acaca* gene, which encodes for the biotin binding site of ACC1. DNA fragments were separated by agarose gel electrophoresis. Presence of a band indicates the successful deletion of Exon 22 in the indicated tissue. One representative picture out of 2 independent experiments with similar results is shown. Following primers were used for DNA amplification, 5'-3' forward: AAGTCCTCAAGGAGCTGGACA; 5'-3' reverse: CCACTGCAATTCAGTCACCATC. (**b**) Representative HE staining of the small intestine (duodenum and ileum) and the distal part of the colon form ACC1$^{lox/lox}$ and ACC1$^{\Delta/\Delta IEC}$ mice 1 day after the last tamoxifen injection. Data representative for 2 independent experiments with n=3-5 mice per group. Bar=200 µm. (**c**) Representative flow cytometry plots illustrating the gating strategy for leukocytes that were isolated from the lamina propria of ACC1$^{lox/lox}$ and ACC1$^{\Delta/\Delta IEC}$ mice on day 9 after 5 consecutive days of tamoxifen treatment. Graphs show frequency (%) of live CD45^{+} T cells (T), B cells (B), dendritic cells (DC), neutrophilic granulocytes (Gr) and macrophages (Mϕ), Data were pooled from 2 individual experiments with n=3-5 mice per group. (**d**) Body weight curve of ACC1$^{lox/lox}$ and ACC1$^{\Delta/\Delta IEC}$ mice treated with tamoxifen for 5 consecutive days (day 0-5) and terminated on day 14. Data is shown as percentage of initial body weight for one representative out of > 3 independent experiments with n = 5 mice per group. Data represent mean and error bars indicate SD (**e**) Effect of long term epithelial ACC1 deletion. Tamoxifen was administered to ACC1$^{lox/lox}$ and ACC1$^{\Delta/\Delta IEC}$ mice for 5 consecutive days at the start of the experiment. Tamoxifen treatment was repeated after

one month for another 5 consecutive days. Representative HE staining of the small intestine (duodenum and ileum) and the proximal and distal parts of the colon form ACC1$^{lox/lox}$ and ACC1$^{\Delta/\Delta IEC}$ mice at 2.5 month (72 days) after the first tamoxifen injection. Data representative for 2 independent experiments with n=3-5 mice per group. Bar=200 µm. Source data are provided as a Source Data file.

Supplementary Figure 2: Reduced frequencies of Lgr5^{high} and Lgr5^{int} progenitors in the colon of ACC1$^{\Delta/\Delta Lgr5}$ mice. (a) DAPI^{-}Epcam^{+}Lgr5^{high} ISCs and DAPI^{-}Epcam^{+}Lgr5^{negative} epithelial cells were isolated from the small intestine of Lgr5-EGFP-IRES-cre^{ERT2} mice. RNA was extracted and qPCR performed for ACC1 (*Acaca*). Data pooled from 3 independent sortings from n=1 mouse per sort (**b**) Frequency of DAPI^{-}Epcam^{+}Lgr5^{high} ISCs and DAPI^{-}Epcam^{+}Lgr5^{int} progenitor cells in crypts isolated from the colon of Lgr5-EGFP-IRES-cre^{ERT2} control and ACC1$^{\Delta/\Delta Lgr5}$ mice 1 day upon the last tamoxifen injection. Data pooled from 2 independent experiments with a total of n= 7 mice per group. Statistical significance was analyzed using unpaired two-tailed Student's t-test with p*<0.05 and **<0.01. Exact p values provided as Source Data. Bar graphs represent mean and error bars indicate SD. Source data are provided as a Source Data file.

Supplementary Figure 3: ACC1 deletion affects expression of genes associated with cellular proliferation and increases cell death in organoids. (**a**) Immunohistochemical staining of Muc2 in organoids. Organoids were prepared of crypts isolated from ACC1$^{\Delta/\Delta IEC}$ and ACC1$^{lox/lox}$ mice. 4-OHT was added for 24h after plating to induce ACC1 deletion in organoids from ACC1$^{\Delta/\Delta IEC}$ mice. After 2 additional days of culture, organoids were harvested, embedded in paraffin and cut into 3 µm sections. Organoid sections were stained using rabbit anti-mucin 2 polyclonal primary antibody (antibodies-online GmbH, Aachen, Germany) and DyLight®594 conjugated donkey anti-

rabbit polyclonal secondary antibody (Abcam, Cambridge, UK, visualized by red color). Nuclear counterstaining (white color) was performed with a mounting medium containing DAPI (Vectashield, Vector Laboratories, USA). Stained tissue sections were examined using a Zeiss Axioskop 40 microscope. Representative pictures are shown from one out of 2 independent experiments with similar results. Bar=50 µm. Red arrows indicate $Muc2^{+}$ cells. (**b, c**) Table showing the top 30 list of most significantly regulated GO terms identified by GSEA (b) and heatmaps of RNA-seq data (c) showing decreased transcript levels of genes associated with DNA replication, cell cycle and chromosome segregation in organoids derived from $ACC1^{\Delta/\Delta IEC}$ mice on day 4 after in vitro ACC1-deletion with 4-OHT (qvalue <0.05). RNA-seq was performed in triplicates from 3 independent experiments. (**d**) Crypts were isolated from $ACC1^{lox/lox}$ and $ACC1^{\Delta/\Delta IEC}$ mice and organoid culture was performed. 4-OHT was added for 24h one day post plating. Organoids were harvested on t day 2 and day 4 after addition of 4-OHT. Apoptosis kit (Invitrogen, 88-8006-74) was used to determine the frequencies of apoptotic and necrotic cells by flow cytometry according to the manufacturer's protocol. Apoptotic cells were defined as $annexinV^{+}7\text{-}AAD^{-}$cells and $annexinV^{+}7\text{-}AAD^{+}$cells as dead/necrotic. Data shown was pooled from 3 independent experiments and analyzed using unpaired two-tailed Student's t-test with *, $p<0.05$. Exact p values provided as Source Data Bar graphs represent mean and error bars indicate SD. Source data are provided as a Source Data file.

Supplementary Figure 4: Impact of ACC1 deletion on main metabolic pathways. (**a**) Seahorse Bioscience XFe96 Analyzer was used to measure oxygen consumption rates (OCR) and extracellular acidification rates (ECAR). Crypts were isolated from the small intestine of $ACC1^{lox/lox}$ and $ACC1^{\Delta/\Delta IEC}$ mice and seeded in 3µl matrigel per well in XF96 cell culture microplates from Agilent Seahorse Bioscience. Organoids were cultured in the presence of 4-OHT

in organoid medium (Mouse IntestiCult™ Organoid Growth Medium, Stemcell Technologies) for 24h, washed and cultured for another 48h in organoid medium before changing to XF DMEM medium (Seahorse bioscience), in which analysis was performed. For the glycolysis test, L-glutamine (Sigma) was added into the medium to a final concentration of 2 mM. Following compounds were loaded to the ports and injected as depicted in the figure: glucose (Sigma) 10mM, oligomycin (Agilent) at 1 μM and 2-DG (Sigma) at 10 mM. For the mitochondrial stress test, glucose (Sigma) was added to a final concentration of 2.5 mM. Analysis of the mitochondrial metabolism was performed by injecting the following compounds as indicated in the figure: oligomycin (Agilent) 2 μM, FCCP (Agilent) at 1 μM and rotenone/antimycin (Agilent) at 0.5 μM. Data was normalized by dividing the OCR or ECAR values of each well by the number of viable organoids counted by microscopy in the respective well. Data shown as mean ±SEM of > 20 replicate wells and are representative of three independent experiments with similar results. (**b-d**) Crypts were isolated from ACC1$^{\Delta/\Delta IEC}$ mice and grown in organoid cultures +/- 4-OHT for 24h to induce ACC1-deletion. (**b**) Acetate (Roth, 6773, at a final concentration of 10 mM) (**c**) PPARγ agonist Troglitazone (abcam, ab141112, at a final concentration of 10 μM) or (**d**) Wnt3a (1324-WN-010, R&D, with the final concentrations of 100 ng/ml) or the GSK-3 inhibitor CHIR99021 (Peprotech, with a final concentration of 5 μM, CHIR) were added to the organoids after removing 4-OHT. On day 5, organoids were imaged and the formation of crypt domains was quantified. More than 20 organoids were counted for each condition. Data was pooled from 3 (**b, c**) or 2 (**d**) independent experiments. Statistical significance was analyzed by One-way ANOVA with Tukey's multiple comparison test with p*<0.05, **<0.01, ***<0.001. ****, p<0.0001. Exact p values provided as Source Data. Boxes in boxplots denote 25th to 75th percentiles with whiskers representing min-max, and the central line the median. Source data are provided as a Source Data file.

Supplementary Figure 5: Epithelial ACC1 deletion does not influence fatty acid uptake and FAO. (**a**) Organoids derived from the small intestine of ACC1$^{lox/lox}$ and ACC1$^{\Delta/\Delta IEC}$ mice were harvested from the culture on day4 after 4-OHT treatment. Organoids were dissociated into single cells using TrypLE (Invitrogen) and incubated with 10 µM BODIPY™ FL C16 (Thermo fisher) at 37 °C for 30 min. Cells were washed with PBS and stained with DAPI prior to analysis of mean fluorescence intensity (MFI) by flow cytometry. Data was pooled from 3 independent experiments. Significance was analyzed using unpaired two-tailed Student's t-test. Exact p values provided as Source Data (**b**) Transcription levels of genes related to FAO. CPM values were derived from RNA-seq of organoids from ACC1$^{\Delta/\Delta IEC}$ and ACC1$^{lox/lox}$ mice at day 4 after 4-OHT treatment. Gene expression levels are shown as fold change relative to the expression level in ACC1-sufficient ACC1$^{lox/lox}$ organoids. RNA-seq was performed in triplicates from 3 independent experiments. Dotted lines indicate a 2-fold change of expression. Bar graphs represent mean and error bars indicate SD. Source data are provided as a Source Data file.

Supplementary Figure 6: IEC-specific ACC1 deletion exaggerates DSS-induced colonic pathology. (**a**) Experimental setting. ACC1$^{lox/ox}$ and ACC1$^{\Delta/\Delta IEC}$ mice were treated with 1.5% (w/v) DSS and 5% (w/v) glucose in their drinking water starting at day 9 after the last tamoxifen treatment. (**b**) Representative H&E staining of the colon shown or one out of 3 independent experiments with a total of n=8-9 mice per group. Bar=200um. (**c**) Histopathological scores of colons based on H&E staining. For histological scoring the presence of rare inflammatory cells in the lamina propria were counted as: 0, increased numbers of inflammatory cells; 1, confluence of inflammatory cells; 2, extending into the submucosa; and 3, transmural extension of the inflammatory cell infiltrate. For epithelial damage, absence of mucosal damage was counted as 0,

discrete focal lymphoepithelial lesions were counted as 1, mucosal erosion/ ulceration was counted as 2, and a score of 3 was given for extensive mucosal damage and extension through deeper structures of the bowel wall. The two subscores were added and the combined histological score ranged from 0 (no changes) to 6 (extensive cell infiltration and tissue damage). Data was pooled from 3 independent experiments with a total of n=8-9 mice per group. Significance was analyzed using unpaired two-tailed Student's t-test with p*<0.05, **<0.01, ***<0.001. ****, p＜0.0001. Exact p values provided as Source Data. Bar graphs represent mean and error bars indicate SD. Source data are provided as a Source Data file.

Supplementary Table 1. qPCR primer sequences.

Gene	Forward primer	Reverse primer
β-actin	TGTTACCAACTGGGACGACA	GGGGTGTTGAAGGTCTCAAA
Lgr5	GACAATGCTCTCACAGAC	GGAGTGGATTCTATTATTATGG
Muc2	GTCCGAAGTGTTACCCTGGA	CCAGGAGTGGAGAAGGTCAG
Lyz1	GGAATGGATGGCTACCGTGG	CATGCCACCCATGCTCGAAT
Tert	TGGGTCTCCCTGTACCAAAT	GGCCTGTAACTAGCGGACACA
Chgb	ACAGGAAGAAGGCAGACGAA	TCCTTCAGTGAAAGGCTCGT
Acaca	GTCTGCTGGGAAGTTAATCCAG	ATCCTGCAGCTCTAGCAGAGG
