# Supplementary material for: Acetyl-CoA-Carboxylase 1-mediated de novo fatty acid synthesis sustains Lgr5+ intestinal stem cell function
Source: Nat Commun. 2022 Jul 9;13:3998. doi: 10.1038/s41467-022-31725-2 (PMC9271096; doi:10.1038/s41467-022-31725-2)
Supplement: Supplementary file 3 — Reporting Summary [file 41467_2022_31725_MOESM3_ESM.pdf]

nature research

Corresponding author(s): Matthias Lochner

Last updated by author(s): April 19th, 2022

Reporting Summary

Nature Research wishes to improve the reproducibility of the work that we publish. This form provides structure for consistency and transparency in reporting. For further information on Nature Research policies, see our Editorial Policies and the Editorial Policy Checklist.

Statistics

For all statistical analyses, confirm that the following items are present in the figure legend, table legend, main text, or Methods section.

n/a	Confirmed	
☐	☒	The exact sample size (*n*) for each experimental group/condition, given as a discrete number and unit of measurement
☐	☒	A statement on whether measurements were taken from distinct samples or whether the same sample was measured repeatedly
☐	☒	The statistical test(s) used AND whether they are one- or two-sided<br>*Only common tests should be described solely by name; describe more complex techniques in the Methods section.*
☐	☒	A description of all covariates tested
☐	☒	A description of any assumptions or corrections, such as tests of normality and adjustment for multiple comparisons
☐	☒	A full description of the statistical parameters including central tendency (e.g. means) or other basic estimates (e.g. regression coefficient) AND variation (e.g. standard deviation) or associated estimates of uncertainty (e.g. confidence intervals)
☐	☒	For null hypothesis testing, the test statistic (e.g. *F*, *t*, *r*) with confidence intervals, effect sizes, degrees of freedom and *P* value noted<br>*Give P values as exact values whenever suitable.*
☒	☐	For Bayesian analysis, information on the choice of priors and Markov chain Monte Carlo settings
☒	☐	For hierarchical and complex designs, identification of the appropriate level for tests and full reporting of outcomes
☒	☐	Estimates of effect sizes (e.g. Cohen's *d*, Pearson's *r*), indicating how they were calculated

Our web collection on statistics for biologists contains articles on many of the points above.

Software and code

Policy information about availability of computer code

Data collection	BD FACS Diva Software (for analysis and sorting)<br>AxioVision and Nuance 2.10.0 software (microscopy)<br>LightCycler 480 Version 1.2.9.11 (qPCR)
Data analysis	GraphPad Prism (v 8.4)<br>FlowJo (v 10.0.7)<br>AxioVision and Nuance 2.10.0 software<br>For RNA-Sequencing:<br>Gene annotation was done by R package “bioMaRt”<br>Differential gene expression was calculated by R package “edgeR”<br>Functional analysis (GSEA) was performed by R package “clusterProfiler”

For manuscripts utilizing custom algorithms or software that are central to the research but not yet described in published literature, software must be made available to editors and reviewers. We strongly encourage code deposition in a community repository (e.g. GitHub). See the Nature Research guidelines for submitting code & software for further information.

Data

Policy information about availability of data

All manuscripts must include a data availability statement. This statement should provide the following information, where applicable:

- Accession codes, unique identifiers, or web links for publicly available datasets
- A list of figures that have associated raw data
- A description of any restrictions on data availability

Sequencing data reported in this paper was uploaded to GEO: Accession number for the RNA-Seq data is GSE188386. Uncropped versions of the western blots shown in Figures 4d, 4h and 5c have been provided as supplementary material. The source data underlying Figs. 1a, 1d, 2a-e, 3b-f, 3h, 4a-c, 4f-g, 4i, 5a, 5b, 5d-f, 6c-e and Supplementary Figs. 1d, 2a, 2e, 3d, 4a-d, 5a, 5,b and 6b are provided as a Source Data file. Other data from the findings of this study are available from the corresponding author upon request.

Field-specific reporting

Please select the one below that is the best fit for your research. If you are not sure, read the appropriate sections before making your selection.

- [x] Life sciences
- [ ] Behavioural & social sciences
- [ ] Ecological, evolutionary & environmental sciences

For a reference copy of the document with all sections, see nature.com/documents/nr-reporting-summary-flat.pdf

Life sciences study design

All studies must disclose on these points even when the disclosure is negative.

Sample size	Sample size for each experiment is indicated in the figure and in the figure legends. No statistical approaches were used in this study to pre-determine the sample size of experiments. We used sample size at least of two or more for each independent experiment, commonly exploited by researchers in the field. Within each independent experiment, at least three technical repicates were analyzed. For experiments including animals, the group size was at least n=3.
Data exclusions	No data was excluded from the analysis.
Replication	At least two to three independent experiments were performed and reproducible results were always obtained and calculated to achieve statistical significance.
Randomization	Samples were chosen randomly without using a specific method. For experiments including animals, sex- and age-matched animals were used for all experiments. Individual animals were randomly distributed among groups without using a specific method.
Blinding	All histopathological scoring and other histological analysis was done by a blined investigator. Analysis of 13C incorporation and RNA-Seqencing was performed by blinded investigators. Organoid cultures and western blot analysis was not blined, as samples had to be arranged according to treatment information.

Reporting for specific materials, systems and methods

We require information from authors about some types of materials, experimental systems and methods used in many studies. Here, indicate whether each material, system or method listed is relevant to your study. If you are not sure if a list item applies to your research, read the appropriate section before selecting a response.

Materials & experimental systems

n/a	Involved in the study	
[ ]	[x]	Antibodies
[x]	[ ]	Eukaryotic cell lines
[x]	[ ]	Palaeontology and archaeology
[ ]	[x]	Animals and other organisms
[ ]	[x]	Human research participants
[x]	[ ]	Clinical data
[x]	[ ]	Dual use research of concern

Methods

n/a	Involved in the study	
[x]	[ ]	ChIP-seq
[ ]	[x]	Flow cytometry
[x]	[ ]	MRI-based neuroimaging

Antibodies

Antibodies used	For flow cytometry:<br>CD24-PE (M1/69), CD11c-APC-eFluor780 (N418), Gr-1-eF660 (RB6-8C5), CD3-PerCP-Cy5.5 (145-2C11), CD19-eF450 (1D3), CD4-PE-Cy7 (GK1.5) and MHC Class II (I-A/I-E)-FITC (M5/114.15) from eBioscience/Thermo Fisher Scientific<br>CD64(FcγRI)-PE (X54-5/7.1) from Biolegend,

	CD45-PE-TexasRed (30-F11) from Invitrogen/Thermo Fisher Scientific<br>For histology:<br>Rabbit anti-OLFM4 (CST, 39141T), rabbit anti-MMP7 (CST, 3801T)<br>For western blot:<br>Rabbit anti-β-catenin (CST, 8814S), rabbit anti-PPARdelta (Thermo Fisher Scientific, PA1-823A), mouse anti-histone H3 (CST, 4499S)
Validation	Rabbit anti-β-catenin (CST, 8814S) has been validated for western blot by the manufacturer and >100 previous publications:<br>https://www.cellsignal.com/products/primary-antibodies/non-phospho-active-b-catenin-ser33-37-thr41-d13a1-rabbit-mab/8814<br>rabbit anti-PPARdelta (Thermo Fisher Scientific, PA1-823A) has been validated for western blot by the manufacturer and 15 previous publications:<br>https://www.thermofisher.com/antibody/product/PPAR-delta-Antibody-Polyclonal/PA1-823A<br>anti-histone H3 (CST, 4499S) has been validated for western blot by the manufacturer > 100 previous publications:<br>https://www.cellsignal.com/products/primary-antibodies/histone-h3-d1h2-xp-rabbit-mab/4499<br>anti-OLFM4 (CST, 39141T) has been validated for histology by the manufacturer and 3 previous publications:<br>https://www.cellsignal.com/products/primary-antibodies/olfm4-d6y5a-xp-rabbit-mab-mouse-specific/39141<br>anti-MMP7 (CST, 3801T) has been validated for histology by the manufacturer and 4 previous publications:<br>https://www.cellsignal.com/products/primary-antibodies/mmp-7-d4h5-xp-rabbit-mab/3801

Animals and other organisms

Policy information about studies involving animals; ARRIVE guidelines recommended for reporting animal research

Laboratory animals	Tamoxifen-inducible intestinal epithelium-specific ACC1-deficient mice were generated by crossing Villin-CreERT2 mice with mice harboring loxP-flanked ACC1 alleles. Tamoxifen-inducible Lgr5-specific ACC1 deficient mice were obtained by crossing Lgr5-EGFP-IRES-CreERT2 with ACC1lox/lox mice. All mice were bred on C57BL/6J background and kept under specific pathogen free condition in individually ventilated cages (IVC) the animal facilities of TWINCORE (Hannover, Germany) or the Helmholtz Centre for Infection Research (Braunschweig, Germany). Animals were bred and maintained at a relative humidity 50±10%, 20–26°C, and in 12 h dark/light cycles (08:00–20:00 light). Food and drinking water was provided ad libitum. For all in vivo experiments, gender-matched 8-14 weeks old age-matched animals were used.
Wild animals	The study did not include wild animals.
Field-collected samples	The study did not include field-collected samples.
Ethics oversight	All animal experiments were performed under the approval by the Lower Saxony Committee on the Ethics of Animal Experiments as well as the responsible state office (Lower Saxony State Office of Consumer Protection and Food Safety) under the permit number 33.9-42502-04-16/2329 and 33.9-42502-04-15/1851

Note that full information on the approval of the study protocol must also be provided in the manuscript.

Human research participants

Policy information about studies involving human research participants

Population characteristics	The study design did not include specifications on the population characteristics.
Recruitment	Samples were collected from adult patients of male and female gender receiving surgery for colon cancer or inflammatory bowel disease. All samples were prepared from healthy parts of the resection. The samples that were used for anlysis were randomly chosen by a blinded investigator, to avoid any bias. Since the study does not include comparisons between groups, it is unlikely that the choice of samples will have any influence on the results.
Ethics oversight	Surgical material from colon tissue was removed at Hannover Medical School. The study design was approved by the local ethics committee (approval number 3082-2016) and each patient has given well-informed written consent. Patents did not receive compensation for their participation.

Note that full information on the approval of the study protocol must also be provided in the manuscript.

Flow Cytometry

Plots

Confirm that:

- [x] The axis labels state the marker and fluorochrome used (e.g. CD4-FITC).
- [x] The axis scales are clearly visible. Include numbers along axes only for bottom left plot of group (a 'group' is an analysis of identical markers).
- [x] All plots are contour plots with outliers or pseudocolor plots.
- [x] A numerical value for number of cells or percentage (with statistics) is provided.

Methodology

Sample preparation	For the isolation of lamina propria cells, the colon and small intestine were isolated from the animals, physically emptied, opened longitudinally, and cut into 2–3cm pieces. Tissue pieces were incubated in PBS containing 30 mM EDTA (Roth) and washed vigorously to remove remaining mucus and to isolate epithelial cells. The tissue was further cut and digested in prewarmed Iscove's modified Dulbecco's medium (Life Tech- nologies/Gibco) containing 1 mg/mL Collagenase D (Roche) and 100 µg/mL DNaseI (Roche). The supernatant was filtered and the remaining tissue was passed through a 100 µm mesh. The cells were separated using a 40%/80% gradient (Percoll solution, GE Healthcare; 900 g, 20 min, 20°C, no break). The interphase was harvested, washed, and cells were subsequently used for flow cytometry analysis.<br>For isolation of mouse intestinal crypt cells, mice were sacrificed and the small intestine was harvested. After removing feces and remaining fat, the small intestine was flushed 3~5 times with ice cold PBS using a syringe with a p200 pipette tip attached. After flushing, a longitudinal incision along the length of the intestine was performed. The intestine was held by one end over a 50 ml falcon tube containing 15 ml of cold PBS with 1% Penicillin/Streptomycin. The intestine was cut into 2 mm pieces, all pieces were collected and transferred into a 50 ml falcon tube. A pre-wetted 10 ml serological pipette was used to wash the intestinal pieces by pipetting up and down 3 times. Tissue pieces were washed 10 times with 15 ml of cold PBS. Afterwards, the tissue pieces were resuspended with 25 ml of Gentle Cell Dissociation Reagent (Stemcell Technologies) and incubated on a rocking platform at 20 rpm for 15 minutes at room temperature. After incubation, the supernatant was carefully removed. The tissue pieces were resuspended in 15 ml of cold PBS + 0.1% BSA, and pipetted up and down 3 times. When the majority of the intestinal pieces settled to the bottom, the supernatant (the first fraction) was transferred and passed through a 70 µm filter into a new 50 ml conical tube. Three additional fractions were obtained. Fractions were centrifuged at 300x g for 5 minutes at 4°C and supernatant was discarded. The pellets containing the isolated crypts were resuspended with 20 mL of cold PBS + 0.1% BSA and were centrifuged at 200x g for 3 min. To obtain single epithelial cells, the crypt suspension was incubated with TrypLE Express (Invitrogen), including Y-27632 (10 µM, Abmole) and N-acetyl-L-cysteine (1 µM, Sigma) for 20 min at 37C. An equal volume of ice-cold DMEM/F-12 was added to the suspension, resuspended and passed through 20 µm filters. Cells were pelleted by centrifugation at 300x g for 10 min.
Instrument	Flow cytometry data was acquired on a LSR II (BD). Cells were sorted on a FACSAria IIu, (Becton Dickinson).
Software	Flow cytomety data was analyzed with FlowJo software (Tree Star).
Cell population abundance	Size of cell populations are visualized by the use of pseudocolor dot plots including outliers.
Gating strategy	Cells were gated on FSC high versus SSC area to gate on singlets. Live cells were gated as LIVE/DEAD Fixable Dead Cell Stain Kit (Life Technologies/Thermo Fisher Scientific) negative cells. For the analysis of lamina propria immune cell populations, cells were further gated on CD45+ and were then further discriminated as T cells (CD3+), B cells (CD19+), Branulocytes (MHCII-, Gr-1+), dendritic cells (MHCII+, CD11c+) or macrophages (MHCII+CD64+). positive gates were selected based on FMO controls.

☒ Tick this box to confirm that a figure exemplifying the gating strategy is provided in the Supplementary Information.
